# Anxiety symptoms and self-reported executive functioning in transgender and gender nonconforming adults: associations with autistic traits and depression

**DOI:** 10.3389/fpsyg.2026.1603606

**Published:** 2026-03-05

**Authors:** Karys Michaela Mossa, Rebecca A. Lundwall, Mikle South

**Affiliations:** 1 Carolina Institute for Developmental Disabilities, School of Medicine, University of North Carolina at Chapel Hill, Chapel Hill, NC, United States; 2 Psychology Department, Brigham Young University, Provo, UT, United States; 3 Emory Autism Center, Emory University School of Medicine, Atlanta, GA, United States

**Keywords:** anxiety, autism, autistic, depression, executive functioning, gender identity

## Abstract

**Background:**

Between 4.8 and 26% of adults presenting to gender dysphoria clinics have autism. Both autistic people and transgender and gender nonconforming (TGNC) people have higher rates of mental health conditions including anxiety and depression and more difficulties with executive functioning, all of which impact quality of life.

**Methods:**

We characterized relationships among mental health, gender nonconformity, and autism in a sample of 54 TGNC individuals, 44.4% of whom were autistic (29.6% formally diagnosed and 14.8% self-identified). We analyzed traits continuously (using the Autism Spectrum Quotient).

**Results:**

Anxiety was the strongest predictor of executive functioning scores, while the contributions of depression symptoms and autistic traits did not rise to the level of statistical significance.

**Conclusion:**

Findings suggest that clinicians can support TGNC autistic people by helping them with their mental health, particularly with managing their anxiety.

## Introduction

Evidence suggests that there is a great deal of overlap between the autistic and the transgender and gender nonconforming communities (TGNC; Glidden et al., 2016; Sala et al., 2020; van der Miesen et al., 2016; Warrier et al., 2020). Studies have shown that somewhere between 5 and 26% of adults presenting to gender dysphoria clinics have an official autism diagnosis in their medical chart (Cheung et al., 2018; Heylens et al., 2018; Kallitsounaki and Williams, 2023). In addition, there are significantly higher rates of self-reported autism diagnoses in the TGNC community regardless of dysphoria compared to cisgender peers (Kristensen and Broome, 2015; Warrier et al., 2020). On a trait level, there is also a trend toward elevated autistic traits in those who are TGNC (Heylens et al., 2018; Jones et al., 2012; van der Miesen et al., 2018; Warrier et al., 2020).

People who are both TGNC and autistic may be especially impacted by concerns that can affect each group separately, such as mental health concerns (e.g., depression and anxiety), social worries and difficulties (e.g., “Who should I share my name and pronouns with and how do I share that information?”), and navigating the care systems (e.g., using executive functioning and social abilities to schedule appointments and attend them). These concerns can amount to exceptional stress (Bowleg, 2012; Crenshaw, 1989).

One approach to understanding this exceptional stress is to use the minority stress model (Hendricks and Testa, 2012; Meyer, 2003). This model distinguishes distal stressors (external events like discrimination) from proximal stress processes (internal vigilance/anticipation, concealment, internalized stigma). In autistic TGNC adults, it is possible that the most relevant executive function (EF) proximal process is hypervigilance/anticipatory threat monitoring (anxiety-like), because it is ongoing and cognitively demanding. Distal stressors “activate” the proximal process. The proximal process is what competes with EF resources in daily life. Minority stress does not merely predict worse mental health; it predicts a specific cognitive-affective state (anxiety/vigilance) that is plausibly the most direct route to EF disruption.

Furthermore, the pathway from anxiety to EF difficulties can be addressed using Attentional Control Theory (Derakshan and Eysenck, 2009; Eysenck et al., 2007). According to this theory, anxiety increases threat/salience monitoring that shifts processing away from goal-directed control (e.g., inhibition, shifting, working memory). Autism is associated with higher baseline cognitive load in social/healthcare navigation (Hill, 2004; St John et al., 2022). Research that combines the study of minority stress and EF has found that minority stress is linked to EF and/or cognitive burden (Nakayama, 2024; Patel et al., 2022).

Drawing these threads together, autistic minority stress and gender minority stress both increase uncertainty, social evaluation of threat, and safety monitoring demands. These are associated with higher baseline cognitive load and make anxiety-driven attentional capture more likely to overwhelm EF resources. Therefore, in autistic TGNC adults, anxiety is not just another symptom; it may be a proximal pathway through which chronic minority stress becomes moment-to-moment EF. Depression is less likely to be the paramount predictor in this model because it is considered downstream “deactivation” whereas anxiety plays the role of ongoing interference that distracts from the resources needed for social navigation and decision-making. Nevertheless, depression has been associated with EF difficulties in meta-analyses (Rock et al., 2014; Snyder, 2013).

### Mental health disparities

While we do not expect depression to be a paramount predictor, research from within both the autistic and the TGNC communities has suggested that both of these groups face many concerns related to their mental health (de Vries et al., 2011; Pitts et al., 2009), as well as barriers to accessing mental health care. Autistic adults have higher rates of all major psychiatric conditions when compared to non-autistic adults (Croen et al., 2015). Some research has shown that autistic adults who are referred for psychiatric care have an average of three current clinical diagnoses, with the most common diagnoses being attention-deficit hyperactivity disorder (ADHD), anxiety disorders, and major depressive disorder (MDD; Joshi et al., 2013). Autistic adults are also at higher risk for suicidal ideation and attempts to die by suicide (Cassidy et al., 2014; Paquette-Smith et al., 2014), and this is likely related to the high co-occurrence of mental health distress.

As with autistic adults, TGNC people are also more likely than their cisgender peers to have co-occurring mental health concerns such as anxiety, depression, eating disorders, psychosis, and substance use disorders (Barr et al., 2021; Reisner et al., 2016). TGNC people are more likely to have suicidal thoughts and to attempt to die by suicide (Grossman and D’Augelli, 2007; Testa et al., 2012). Though there has not been a great deal of research on the mental health of people identifying as both autistic and gender nonconforming, some evidence suggests autistic people who identify as TGNC or who are experiencing gender dysphoria have worse overall mental health than cisgender autistic people (George and Stokes, 2018; Murphy et al., 2020).

It is important to note that these disparities in mental health are likely related in part to poor treatment of autistic and TGNC people by society. Importantly, anticipation of discrimination appears to drive care avoidance (Kcomt et al., 2020). Both autistic (Gibbs et al., 2022; Weiss and Fardella, 2018) and TGNC (Griner et al., 2020; Marx et al., 2021) people are stigmatized and viewed negatively by peers and are often bullied and victimized because of their differences. Discrimination has a well-established connection with mental health disparities in people of color (Carter et al., 2017; Schmitt et al., 2014) as well as for sexual and gender minorities (Hendricks and Testa, 2012; Meyer, 2003). Evidence suggests that poor mental health in the autistic population is related to discrimination and minority stress in the same ways that it is related in other minoritized groups (Botha and Frost, 2020). As such, the complex trauma of repeated discrimination based on gender expression and autistic traits likely contributes to the development of mental health concerns for those reporting gender nonconformity and neurodivergence.

### Executive functioning

As mentioned previously, the anxiety that minority group members feel can lead to problems with EF. Executive functioning is a collection of cognitive processes that assist with the top-down control of thoughts and behaviors (Jones et al., 2012). It encompasses abilities related to guiding, directing, and managing cognitive, emotional, and behavioral functions. Executive functioning is related to adaptive functioning (Gilotty et al., 2002), which is the ability to perform daily tasks and to care for oneself. Thus, executive functioning skills are important for navigating the world and doing things like keeping track of tasks, completing tasks, weighing options, making decisions, and navigating the social world efficiently. Accordingly, stronger EF skills are associated with better quality of life, including improved ability to build and maintain relationships and to maintain and care for physical and mental health (de Vries and Geurts, 2015; Dijkhuis et al., 2017). Within this context, anxiety is one pathway through which EF may be affected.

Beyond anxiety alone, broader social mistreatment may also contribute to EF difficulties. When a person has intersecting marginalized identities they are trying to navigate, or they are attempting to manage tasks related to those identities that do not come naturally, their coping systems may be overwhelmed (Schmitt et al., 2014). This idea has been modeled by Hendricks and Testa (2012) and adapted by Botha and Frost (2020). When coping systems are taxed in this way, EF challenges may become more apparent, potentially negatively impacting quality of life.

At the same time, EF difficulties are not specific to minority stress; they are also common in a range of clinical conditions (e.g., ADHD, depression, anxiety) and may be comorbid for genetic and/or other environmental reasons. A meta-analysis by Snyder (2013) reports on differential effects on EF in adult clinical populations (primarily major depressive disorder). Notably, researchers have found these mental health conditions in both the autistic (Hollocks et al., 2019) and TGNC communities (Pinna et al., 2022).

This overlap matters for interpretation of EF findings in autism. It is difficult to study overall executive functioning in autistic people, as results from many studies have been inconsistent in demonstrating strengths and challenges in executive functioning for autistic people (St John et al., 2022). These findings may be unclear due to a lack of understanding of the impact of co-occurring mental health concerns and stressors, including gender dysphoria and navigating the gender-affirming medical care system or managing mental and physical health concerns. However, one clearer pattern is that autistic adults’ EF performance worsens under increased cognitive load (Bodner et al., 2019; Geurts and Vissers, 2012). This may have particular relevance for autistic TGNC individuals, as navigating an unwelcoming society as transgender or gender nonconforming can be a burdensome cognitive task that consistently co-occurs with other everyday cognitive demands (St John et al., 2022).

However, these autism-focused findings and interpretations point to a broader question: whether analogous “load” mechanisms operate in TGNC populations more generally. Addressing this, Strang et al. (2022) developed a model of executive functioning in TGNC people that extends the gender minority stress model (Hendricks and Testa, 2012) by specifying how gender-related stressors and identity-related demands may tax EF resources. This model follows the gender minority stress model (Hendricks and Testa, 2012), and states that TGNC people have additional demands on their executive functioning capacities as a result of their gender identity. It also suggests that factors such as having higher levels of autistic traits and internalizing symptoms may overwhelm already taxed executive functioning capacities in TGNC people, because of the high degree of overlap between identifying as TGNC and being autistic or having a co-occurring internalizing mental health condition, such as depression or anxiety. Strang et al.'s (2022) stated in their model that affirming environments that help meet TGNC people’s needs, and the use of gender-affirming care, may also impact executive functioning by reducing the demand for executive functioning capacities.

While Strang et al.'s (2022) model posits that internalizing symptoms may overwhelm EF capacities in TGNC youth, it does not delineate the potential differential unique contribution of anxiety versus depression. Building on the minority stress framework extended to autism (Botha and Frost, 2020), we hypothesize that anxiety may constitute a more direct and acute cognitive load on EF resources than depression in this population. Anxiety is characterized by hyper-vigilance and future-oriented threat appraisal-processes that continuously consume attentional and working memory resources critical for EF. In contrast, depressive symptoms, often marked by psychomotor retardation and anhedonia, may impact motivation and initiation more than the core planning/organizing aspects of EF. For TGNC autistic adults, navigating societal stigma, gender identity disclosure decisions, and complex healthcare systems are chronic, anxiety-provoking demands that likely place a sustained tax on EF systems. Therefore, we predict that in our sample of TGNC adults, self-reported anxiety will be the strongest independent predictor of EF difficulties, surpassing the contributions of depressive symptoms and autistic traits.

### Rationale for the present study

Prior work suggests that TGNC and autistic adults experience elevated internalizing symptoms and executive functioning challenges. Building on minority stress and attentional control theory, we tested whether anxiety would show the strongest independent association with executive functioning difficulties relative to depressive symptoms and autistic traits.

### Research questions

In TGNC adults, what is the relative contribution of anxiety symptoms, depressive symptoms, and autistic traits to self-reported executive functioning difficulties? Hypotheses. Based on minority stress and attentional control theory, we hypothesized that anxiety would account for the largest unique variance in executive functioning difficulties when modeled alongside depression and autistic traits. We further expected autistic traits to exacerbate the association between internalizing symptoms and executive functioning.

## Method

### Participants

Because TGNC research often samples from gender dysphoria clinics, it may underrepresent individuals who do not experience sufficient distress to seek specialty care or who face barriers to access. To enhance representativeness and capture a broader range of experiences, we employed community-based recruitment strategies, including social media platforms, public forums, community flyers, word-of-mouth referrals, and outreach through queer resource organizations in Utah. This approach yielded a sample of 54 TGNC adults (aged 18 + years, English-speaking) who self-identified as TGNC. We retained self-diagnosed autistic participants given evidence for the validity for this approach comparable to clinical diagnosis in community samples and the number of significant barriers to receiving formal, clinical diagnosis as an adult (Ardeleanu et al., 2025; McDonald, 2020; Overton et al., 2024). This is especially important for transgender and gender diverse adults who experience additional barriers to diagnosis and acceptance, where exclusion risks systematic underrepresentation of people already marginalized by existing diagnostic systems (Ardeleanu et al., 2025; Adams et al., 2025). Consistent with minority stress theory and prior literature, we anticipated elevated anxiety among participants (Botha and Frost, 2020; Hendricks and Testa, 2012; Meyer, 2003).

### Procedure

Data analyzed for this paper were collected as part of a larger study [redacted]. We collected data online via a self-report Qualtrics (Provo, UT) survey including the measures listed below. We asked interested participants to reach out to the research team via email, where they were provided with instructions for participation and a link to the survey. Participants were then asked to email the team after completing the survey to receive the link to the secondary survey for entry into a drawing for a $50 Amazon gift card (chances of winning were 1 in 25). All participants completed the same measures, regardless of their recruitment origin or demographic factors.

### Measures

#### Demographics questionnaire

Survey participants completed a demographics questionnaire asking about sex assigned at birth, gender identity, sexual orientation, relationship status, race, ethnicity, education, work, socio-economic status, languages spoken, religion, disability status, and mental health diagnoses (Supplementary Appendix A).

#### Gender affirmation history

Survey participants completed a questionnaire regarding social and medical transitioning. This questionnaire asked about social transition steps such as coming out, name and pronoun changes, gender expression changes via grooming and dress, and voice therapy. It also asked about medical transition steps such as puberty suppression, gender-affirming hormones, and gender-affirming surgical procedures (Supplementary Appendix B).

#### Autism Spectrum Quotient

Survey participants completed the Autism Spectrum Quotient (AQ; Baron-Cohen et al., 2001), which is a standardized self-report measure of autistic traits. The test–retest reliability for the AQ has been found to be acceptable and the internal consistency for all domains has been shown to be moderate to high (Baron-Cohen et al., 2001). One examination found that the AQ can discriminate autistic traits in samples with elevated levels of autistic traits over non-autistic controls with only two of the items scoring higher in the control sample (Woodbury-Smith et al., 2005). For the original version, the suggested cut-off score is 32 for a clinically significant level of autistic traits, but we measured autistic traits on the AQ dimensionally, using it as a continuous variable for autistic traits. We used the Autism Spectrum Quotient (AQ) total score as a continuous measure of autistic traits in primary analyses. We did not include formal autism diagnosis status as a covariate in the regression model to avoid multicollinearity with the AQ and to focus on the dimensional relationship of traits to EF.

#### Depression

Survey participants completed the Beck Depression Inventory – 2nd Edition (BDI-II; Beck et al., 1996), which is a standardized self-report measure of depressive symptoms in the past 2 weeks. The BDI-II is a widely used indicator of depressive symptom severity aligned with the diagnostic criteria for depressive disorders in the DSM-IV (American Psychiatric Association, 2000). It includes questions related to feelings of sadness, pessimism, failure, loss of pleasure, suicidal thoughts, loss of interest, feelings of worthlessness, irritability, fatigue, and other related depressive symptoms. Its reliability has been shown to be in the excellent range, and its internal consistency is considered adequate (Arbisi and Farmer, 2001).

#### Anxiety

Survey participants completed the Beck Anxiety Inventory (BAI; Beck et al., 1988), which is a standardized self-report measure of anxiety symptoms over the past week. It includes questions assessing both somatic (e.g., numbness and tingling, difficulty breathing, or heart pounding or racing, shaky) and thought-related (e.g., unable to relax, terrified, nervous, fear of losing control) anxiety symptoms. The BAI is a widely used measure of anxiety symptom severity, and its internal consistency and test–retest reliability are both classified as excellent (Dowd and Waller, 1998).

#### Executive functioning

Survey participants completed the self-report version of the Behavior Rating Inventory of Executive Function – Adult Version (BRIEF-A; Roth et al., 2005). The BRIEF-A is a standardized measure of the participants’ perspective on their own executive functioning and self-regulation in their day-to-day life. We used the self-report version of this measure. There are nine clinical scales on the BRIEF-A: emotional control, inhibit, initiate, organization of materials, plan/organize, self-monitor, shift, task monitor, and working memory. It also provides two broad indices of behavioral regulation and metacognition, as well as an overall summary score. The BRIEF-A has been shown to have high reliability, validity, and clinical utility across a large normative sample and across people with a range of conditions that impact executive functioning (Roth et al., 2005).

### Community involvement

The lead author of this paper is a queer autistic researcher and clinician who completed this project as part of a dissertation in pursuit of her doctoral degree. Additionally, prior to data collection, the research team sought feedback online from people who identify as both autistic and TGNC about the content and format of the survey used to collect data for this project. We integrated this feedback into the final version of the survey that was used for data collection.

### Data analysis

Due to the small sample size, we retained all participants. However, missing data was small (three participants were missing “income,” one was missing “sex assigned at birth” [preferred not to specify], and one was missing “autism diagnosis”). One value was more than 1.5 interquartile ranges above the third quartile. We used SPSS (IBM Corp, 2022) for all statistical analyses.

## Results

### Demographics

#### Age, race, sex assigned at birth, gender identity, sexual orientation, and relationship status

Participants were 18–56 years old (*M* = 26.91, *SD* = 8.77). Full distributions for race, sex assigned at birth, gender identity, sexual orientation, and relationship status are presented in Table 1. Briefly, the sample was predominantly White with substantial nonbinary representation, and most participants were not in a relationship.

**Table 1:** Personal demographics.

Race	*N*	%
White	39	72
Black	3	6
Middle Eastern/North African	1	2
Multiracial	11	20
Sex assigned at birth	*N*	%
Female	32	60
Male	19	34
Intersex	2	4
Preferred not to disclose	1	2
Gender identity	*N*	%
Female	18	33
Male	8	15
Nonbinary	20	37
Undisclosed or Uncertain/Exploring	8	15
Sexual orientation	*N*	%
Straight/Heterosexual	5	9.3
Gay or Lesbian	11	20.4
Bisexual	17	31.5
Pansexual	4	7.4
Asexual	5	9.3
Queer	4	7.4
Uncertain/Exploring	4	7.4
Other	4	7.4
Relationship status	*N*	%
Not in a relationship	34	63
Married	7	13
Cohabitating with a partner	7	13
Divorced	1	1.9
Other	5	9.3

#### Work status, school status, and education level

Regarding education and employment, 39% were not working, 61% were not currently enrolled in higher education, and 46% had completed a bachelor’s degree or higher (with many others reporting some college). It is unclear how these demographics compare to the broader TGNC population, as findings on educational attainment in LGBTQ+ samples are mixed (Black et al., 2000; Ueno et al., 2013). Within TGNC populations, educational outcomes may vary by timing of transition milestones and related school-based social impacts (Wilkinson et al., 2018), and could also reflect minority stress, though this has not been directly tested.

#### Income and financial situation

Participants were distributed across income brackets, with most reporting sufficient funds for necessities (and, for many, some discretionary spending). This can be compared to research from the Human Rights Campaign based on the 2019 Behavioral Risk Factor Surveillance System (BRFSS) data suggest that 29% of transgender adults live in poverty, as compared to 16% of cisgender and straight adults (Human Rights Campaign, n.d.; Human Rights Campaign, 2023; Ipsos, 2018; McDonald, 2020; Mallory and Redfield, 2023). The income level of our sample may be related to the race of the majority of our participants being White.

#### Disability status, autism diagnoses, medical and mental health diagnoses, and suicidality

Most participants reported at least one disability, with psychiatric and neurodevelopmental conditions most common. Regarding autistic identity, 43% identified as autistic (formally diagnosed or self-identified) and an additional subgroup reported exploring diagnosis/identity. On the BDI-II suicidality item, most denied suicidal thoughts, while a subset endorsed passive thoughts without intent and a smaller subset endorsed more serious ideation; resources were provided to all participants.

Gender affirmation and gender-affirming care history are summarized in Table 2. Briefly, social transition experiences were common, whereas puberty suppression and surgical history were less common in this sample.

**Table 2:** Gender affirmation demographics.

Puberty suppression medication	*N*	%
Have used puberty blockers	2	3.7
Have not used puberty blockers	52	96.3
Gender-affirming hormone therapy	*N*	%
Have used gender-affirming hormones	28	52
Have not used gender-affirming hormones	26	48
Surgical transition aspects	*N*	%
Have had at least one gender-affirming surgical procedure	8	15
Have not had any gender-affirming surgical procedures	46	85
Social transition aspects	*N*	%
Have engaged in social transition	45	83.3
Two social transition aspects	2	4.4
Three social transition aspects	6	13.3
Four social transition aspects	4	8.9
Five social transition aspects	18	40
Six social transition aspects	10	22.2
Seven social transition aspects	5	11.1
Have not engaged in social transition	9	20

For social transitions aspects, the percentages for rows two through seven are based on the 45 participants who indicated social transition.

### Descriptive statistics

Within our sample, 46.3% of participants had a T-score on the BRIEF-A that was above the cut score indicating clinical concern (i.e., 65). For the BDI-II, 53.7% of participants had a total score of 20 or higher, indicating moderate levels of depressive symptoms. For the BAI, 55.6% of participants had a total score of 16 or higher, indicating moderate levels of anxiety symptoms. On the AQ, 53.7% of participants’ total scores were above the cut score of 32 which indicates significantly elevated autistic traits (See Table 3 for more details).

**Table 3:** Descriptive statistics for measures.

Measure	*M*	SD	Range	Cut scores
BRIEF-A (T-scores)	62.61	14.95	34–99	65
BDI-II (Total scores)	21.00	14.10	0–59	20
BAI (Total scores)	22.11	12.58	0–61^1^	16
AQ (Total scores)	29.28	9.11	12–45	32

The BRIEF-A scores consist of the Global Executive Composite. ^1^ The person with a score “61” was the only outlier on any of the measures. An outlier was defined as more than 1.5 interquartile ranges above the third quartile.

### Main regression analysis

We hypothesized that within our sample, executive functioning as measured by the BRIEF-A would be impacted by mental health functioning (i.e., anxiety and depression) as measured by the BDI-II and the BAI, and autistic traits as measured by the AQ. We entered these variables into a regression simultaneously, with executive functioning as the outcome variable. We did not enter autism diagnosis (formal or self-diagnosis) into the regression.

We report unstandardized coefficients (B) with standard errors and standardized coefficients (*β*). The overall model was significant *R^2^* = 0.48, (*p* < 0.001). Anxiety was the only predictor that contributed significantly to the model based on our alpha of 0.05 (see Table 4).

**Table 4:** Regression analysis summary for autistic traits, anxiety, and depression predicting executive functioning, *N* = 54.

Variable	*B*	*SE*	*β*	*t*	*p*	LL 95%CI for β	UL 95%CI for β
(Constant)	37.35	5.44		6.87	<0.001		
Anxiety (BAI)	0.53	0.156	0.45	3.43	0.001	−0.09	0.99
Depression (BDI-II)	0.26	0.141	0.22	1.83	0.073	−0.04	0.48
Autistic traits (AQ)	0.31	0.172	0.19	1.81	0.077	−0.12	0.50

The overall model was significant *R*^2^ = 0.48, *p* < 0.001.

Depression (as expected according to Derakshan and Eysenck, 2009; Eysenck et al., 2007) did not reach significance in the model. However, depression and AQ showed imprecise estimates with *p*-values from 0.073–0.077. Larger samples are needed to estimate these effects more precisely.

### Sensitivity analysis

In the full sample, anxiety showed a robust unique association with EF (*β* = 0.45, *p* = 0.001). Depression and autistic traits were not statistically significant at *α* = 0.05; however, estimates were imprecise and the evidence was inconclusive (*p*s = 0.073–0.077). When analyses were restricted to participants with a formal ASD diagnosis (*n* = 15), the model and predictors are not statistically significant. Estimates differed in magnitude in the diagnosed subgroup, but confidence intervals were wide and conclusions about subgroup differences are not warranted (Table 5).

**Table 5:** Regression analysis summary for autistic traits, anxiety, and depression predicting executive functioning for those reporting a formal ASD diagnosis.

Variable	*B*	*SE*	*β*	*t*	*p*	LL 95%CI for *β*	UL 95%CI for *β*
(Constant)	37.32	15.40		2.42	0.03		
Anxiety (BAI)	0.11	0.33	0.10	0.33	0.75	−0.01	0.21
Depression (BDI-II)	0.20	0.26	0.23	0.77	0.46	0.03	0.43
Autistic Traits (AQ)	0.67	0.44	0.39	1.50	0.16	−0.27	1.05

*N* = 15. The overall model was significant *R*^2^ = 0.32, *p* = 0.22.

## Discussion

### Main findings

This study is the first of its kind to examine these questions in an adult, TGNC, autistic sample recruited from the general population. We examined how anxiety, depression, and autistic traits relate to executive functioning (EF) in an adult TGNC sample with substantial autistic identification recruited from the general population. The primary finding was that anxiety showed the clearest unique association with EF, whereas depression and autistic traits were not statistically significant predictors in the main model, though both may have been difficult to detect with the small sample size.

When analyses were limited to participants with a formal ASD diagnosis (*n* = 15), effects were generally in the same direction but were not statistically detectable and had wide uncertainty, consistent with limited power; thus, the subgroup analysis does not provide strong evidence that associations differ by diagnostic status.

The lack of a statistically detectable association between autistic traits and EF is framed as plausible given mixed findings in the autism–EF literature: some studies report EF difficulties in autistic adults (St John et al., 2022), whereas others show relative strengths in certain EF domains (Geurts et al., 2009). The discussion highlights evidence that higher cognitive load can worsen EF in autistic people (Bodner et al., 2019; Geurts and Vissers, 2012), and it links this to the study’s conceptual rationale for expecting autistic traits to matter for EF, drawing on models in which anxiety disrupts cognitive performance (Derakshan and Eysenck, 2009; Eysenck et al., 2007). Specifically, the authors propose that the sustained cognitive demands of navigating TGNC-related stressors (e.g., monitoring safety, anticipating social reactions, managing complex gender-affirmation systems) may elevate anxiety, which then more directly interferes with EF (e.g., working memory, cognitive flexibility). Autistic traits (e.g., sensory sensitivities, social-communication differences) may intensify this cognitive burden, even if they do not emerge as an independent predictor of EF in this dataset.

### Clinical implications

Clinically, the findings suggest that when TGNC autistic people present with EF-related difficulties, interventions may be most effective if they prioritize anxiety reduction rather than presuming autism-linked EF impairment. This is particularly relevant given the substantial real-world planning and organization demands involved in gender affirmation tasks (Strang et al., 2022). If anxiety and vigilance are proximal pathways from minority stress to EF problems, then approaches that reduce anxiety (e.g., CBT targeting catastrophic thinking and hypervigilance) and supports that reduce stressor exposure (e.g., gender-affirming environments and care-navigation assistance) may indirectly improve everyday EF and access to gender-affirming care (Strang et al., 2022).

### Limitations and future directions

A primary limitation is sample size, which constrained statistical power to detect smaller-to-moderate effects and limited confidence in subgroup inferences. Relatedly, diagnostic certainty was limited because autism diagnosis status was self-reported and not clinician-verified; accordingly, conclusions about “autistic” TGNC adults should be interpreted as applying to a broad range of participants (e.g., experiencing diagnostic exploration, self-identifying as autistic, or self-reporting formal diagnosis) rather than those with a confirmed ASD. The study also relied on a dimensional trait measure (AQ) that may be inflated or confounded by current internalizing symptoms, as far as anxiety and depression can shape social functioning and self-perception; thus, AQ scores may partially reflect symptom burden rather than autistic traits alone (Ashwood et al., 2016). Finally, the exclusive use of self-report measures (including EF) and the inability to adjust for key covariates—especially ADHD, given its high comorbidity and strong links to EF—may have introduced measurement and omitted-variable bias that could attenuate or distort observed associations.

Contextual factors also limit generalizability. Because TGNC experiences vary substantially by sociopolitical context, the broad geographic recruitment and heterogeneous participant environments likely introduced meaningful, unmeasured variability tied to local attitudes and structural protections (Bränström and Pachankis, 2021; Hammond and Moretti, 2025; Worthen et al., 2017; Falck and Bränström, 2023; Williamson, 2023). In addition, data collection occurred amid a period of heightened anti-transgender legislation and rhetoric in the United States, which may have elevated distress and anxiety in ways that are time-specific and could influence both mental health and EF-relevant self-reporting (Houghtaling et al., 2025; Santhanam, 2023).

## Conclusion

This study provides information to researchers and clinicians in this field about how executive functioning, autistic traits, anxiety, and depression are related to one another in those reporting TGNC and autism. This study provides support for the theory that anxiety is associated with EF in autistic people, just as it does in neurotypical people. It is important for clinicians to remember though that both TGNC and autistic people have a higher probability of being anxious and depressed than cisgender and neurotypical people, so the impact on executive functioning may be related to those mental health concerns. As such, rather than placing a focus on managing executive functioning challenges directly, a focus on managing anxiety symptoms in a therapy context may alleviate some of the EF burden placed on autistic TGNC clients by the social and medical systems they need to navigate for transitioning. Further work is needed in this area to confirm this finding and to look into these questions with a larger sample that is powered to detect effects at these intersections.

## Data Availability

The datasets presented in this article are not readily available because the population is small enough that sharing the data might unintentionally identify participants who wish to remain anonymous. Requests to access the datasets should be directed to karys.mossa@cidd.unc.edu.
