# Supplementary material for: Anxiety symptoms and self-reported executive functioning in transgender and gender nonconforming adults: associations with autistic traits and depression
Source: Front Psychol. 2026 Mar 5;17:1603606. doi: 10.3389/fpsyg.2026.1603606 (PMC12999928; doi:10.3389/fpsyg.2026.1603606)
Supplement: Supplementary file 1 [file Supplementary_file_1.docx]

**APPENDIX A**

**Demographics Questionnaire**

1. In what month and year were you born? (please use MM/YYYY format)
2. What sex were you assigned at birth, on your original birth certificate?
   1. Male
   2. Female
   3. Intersex
   4. Not listed (please specify)
3. What is your current gender identity? How do you describe yourself?
   1. Male / Man or Trans Male / Man
   2. Female / Woman or Trans Female / Woman
   3. Gender-diverse (nonbinary, genderfluid, genderqueer, gender nonconforming, bigender, agender, two-spirit)
   4. Not sure (exploring my gender identity)
   5. Not listed (please specify)
4. How strongly to you identify as male or female?
   1. Male
   2. Male-leaning
   3. Neutral (equally neither or both)
   4. Female-leaning
   5. Female
5. How would you describe your identity class?
   1. Male cisgender
   2. Male transgender
   3. Male-leaning cisgender
   4. Male-leaning transgender
   5. Neutral cisgender
   6. Neutral transgender
   7. Female-leaning cisgender
   8. Female-leaning transgender
   9. Female cisgender
   10. Female transgender
6. What are your pronouns?
   1. They/them
   2. He/him
   3. She/her
   4. Neopronouns (e.g., Xe / Xir / Xem, etc.)
   5. Other
7. What is your sexual orientation?
   1. Heterosexual / Straight
   2. Gay or Lesbian
   3. Bisexual
   4. Pansexual
   5. Asexual
   6. Queer
   7. Not sure (exploring my sexual orientation)
   8. Not listed (please specify)
8. What is your relationship status?
   1. Single
   2. Married
   3. Civil union
   4. Cohabitating
   5. Separated
   6. Divorced
   7. Widowed
   8. Not listed (please specify)
9. Although the categories listed below may not represent your full identity or use the language you prefer, for the purpose of this survey, please indicate which group(s) below most accurately describes your racial identification? (You may select more than one option)
   1. Asian
   2. Black
   3. Latinx / Latine / Hispanic
   4. Middle Eastern / North African
   5. Pacific Islander / Native Hawaiian
   6. White
   7. Multiracial
   8. Not listed (please specify)
10. Multiracial people can identify in various ways. For example, some people identify with a specific racial heritage, and some identify as “multiracial.” Please describe the race with which you primarily identify. Please also describe any other races that are part of your identity.
11. Are there any other words you would use to describe your race, ethnicity, or culture?
12. What languages do you currently speak?
13. How fluent are you currently in English?

0 = not fluent at all, 5 = moderately fluent, 10 = completely fluent

1. What language is currently used in your home most of the time?
2. What is the highest grade in school, year in college, or post-college degree work have you completed?
   1. Did not finish high school (please enter highest grade completed)
   2. High school diploma, GED, or equivalent degree
   3. Some college credit (no degree completed)
   4. Technical / Trade / Vocational training
   5. Associate degree
   6. Bachelor’s degree
   7. Master’s degree
   8. Doctorate degree
3. Are you currently a student?
   1. Part-time student
   2. Full-time student
   3. Not a student
4. Are you currently involved in paid work?
   1. Not at all
   2. Working 1-20 hours per week
   3. Working 21-30 hours per week
   4. Working 31-40 hours per week
   5. Working over 40 hours per week
5. What is your current job title?
6. Currently, your total household income (all earners) is:
   1. $0-$15,000
   2. $15,001-$25,000
   3. $25,001-$35,000
   4. $35,001-$50,000
   5. $50,001-$75,000
   6. $75,001-$100,000
   7. $100,001-$200,000
   8. More than $200,000
7. What is the total number of people who rely on this income (including yourself)?
   1. 1
   2. 2
   3. 3
   4. 4
   5. 5
   6. 6
   7. 7
   8. 8
   9. 9
   10. 10+
8. Currently, how would you describe the financial situation of your family?
   1. Routinely unable to purchase sufficient food or other necessities.
   2. Occasionally unable to purchase sufficient food or other necessities.
   3. Have enough money for necessities.
   4. Have enough money for necessities and some luxuries.
9. With what religion or spiritual practice (if any) do you currently identify? (You may choose more than one option)
   1. Christian – Catholic
   2. Christian – Protestant (examples: Baptist, Episcopal, Methodist, Evangelical, Lutheran, Quaker, etc.)
   3. Christian – LDS/Mormon
   4. Christian – Other
   5. Jewish
   6. Muslim
   7. Hindu
   8. Buddhist
   9. Spiritual but not religious
   10. Agnostic
   11. Atheist
   12. Other (please specify)
10. Do you have a disability? (You may select more than one option)
    1. No disability
    2. Acquired / traumatic brain injury
    3. Blind / low vision
    4. Deaf / hard of hearing
    5. Cognitive, developmental, or learning disability (please specify)
    6. Chronic illness / medical condition (please specify)
    7. Mental illness (please specify)
    8. Physical / mobility concern that affects walking (please specify)
    9. Physical / mobility concern that does not affect walking (please specify)
    10. Speech / communication condition (please specify)
    11. Other (please specify)
11. Do you have an autism spectrum condition diagnosis? (Examples: autism, autism spectrum disorder, autistic disorder, Asperger's, pervasive developmental disorder)
    1. No
    2. Yes, I have been formally diagnosed. (please specify diagnosed condition)
    3. Yes, I am self-diagnosed.
    4. I believe I may be autistic but am exploring and have not been diagnosed.
12. Do you have any other physical or mental health concerns that have not been discussed in previous questions?
    1. No
    2. Yes (please specify)
13. Think of the options below as a ladder representing where people stand in our society. At the top of the ladder are the people whose social class (income level, occupation, and education level) is the most ideal, accepted, and valued in our society. At the bottom of the ladder are the people whose social class is the least ideal, accepted, and valued in our society. The higher up you are on this ladder, the closer you are to the people at the very top and the lower you are, the closer you are to the bottom. Where would you put yourself on the ladder? Please select the option where you think you stand.

10 – most ideal, valued, accepted social class

9

8

7

6

5

4

3

2

1 – least ideal, valued, accepted social class

**APPENDIX B**

**Gender Affirmation History Questionnaire**

The following questions are related to social and medical transition steps that you may or may not have taken. We wish to note that we ask these questions for clarification and that we do not believe these questions are not indicative of your validity in your gender identity.

1. Have you taken any steps to socially transition? (examples: coming out, changing name and pronouns, changing gender expression)
   1. No
   2. Yes
2. Which of the following steps have you taken? (You may select more than one option)
   1. Coming out to family / friends
   2. Changing name socially or legally
   3. Changing pronouns
   4. Changes in dress or grooming for gender expression
   5. Binding / packing / tucking / padding
   6. Changing participation in gendered activities (examples: living arrangements, sports, gendered clubs, etc.)
   7. Voice and communication or speech therapy to match vocal characteristics with gender identity
   8. Other (please specify)
3. When did you start the process of socially transitioning? (Please use MM/DD/YYYY format. If you are not certain, give your best guess).
4. Have you ever taken puberty suppression medication (puberty blockers)?
   1. No
   2. Yes
5. When did you start taking puberty blockers? (Please use MM/DD/YYYY format. If you are not certain, give your best guess.)
6. Are you currently taking puberty blockers?
   1. Yes
   2. No
7. When did you stop taking puberty blockers? (Please use MM/DD/YYYY format. If you are not certain, give your best guess.)
8. Have you ever taken gender-affirming hormones?
   1. No
   2. Yes
9. When did you start taking hormones? (Please use MM/DD/YYYY format. If you are not certain, give your best guess.)
10. Are you currently taking hormones?
    1. Yes
    2. No
11. When did you stop taking hormones? (Please use MM/DD/YYYY format. If you are not certain, give your best guess.)
12. Have you had any gender affirming surgical procedures?
    1. Yes
    2. No
13. Which of the following gender affirming surgical procedures have you had? (You may select more than one option)
    1. MtF breast / chest / top surgery: augmentation mammoplasty (implants/lipofilling)
    2. FtM breast / chest / top surgery: subcutaneous mastectomy/creation of a male chest
    3. MtF genital / bottom surgery: penectomy, orchiectomy, vaginoplasty, clitoroplasty, vulvoplasty
    4. FtM genital / bottom surgery: hysterectomy/salpingo-oophorectomy, urethral reconstruction, metoidioplasty, phalloplasty, vaginectomy, scrotoplasty, implantation of erection prosthesis, implantation of testicular prostheses
    5. MtF other surgery: facial feminization, liposuction/lipofilling, voice feminization surgery, thyroid cartilage reduction, gluteal augmentation, hair reconstruction
    6. FtM other surgery: voice masculinization surgery, liposuction/lipofilling, pectoral implants
    7. Other procedures (please specify)
14. When did you have your first gender affirming surgical procedure? (Please use MM/DD/YYYY format. If you are not certain, give your best guess.)
15. Is there anything else you would like us to know about your gender affirmation process?
